# Emotion Processing and the Role of Compassion in Psychotherapy from the Perspective of Multiple Selves and the Compassionate Self

**DOI:** 10.1155/2019/7214752

**Published:** 2019-01-06

**Authors:** Kenichi Asano

**Affiliations:** Mejiro University, 4-31-1 Nakaochiai, Shinjuku-ku, Tokyo 161-8539, Japan

## Abstract

Emotion processing is an important factor for successful psychotherapy. Clients tend to suffer from maladaptive emotions, which contribute to states of confusion, rumination, and stagnation. The therapist should demonstrate adequate empathy and understanding of the client's complaints to help the client to recognize and respect their own emotions and desires. In most cases, there is more than one desire, and each desire should be confronted. The compassionate self exercises are helpful to distinguish and integrate confused states. In this report, the author introduces a case in which the therapist helped a client to process emotional experiences by leading the client to pay attention to her own emotional responses. The client accessed multiple desires for each emotion and recognized the context for each. To integrate multiple desires and contexts, the therapist used multiple selves exercises from Compassion Focused Therapy. The compassionate self exercises play a role in integrating complicated emotions and in directing the client toward making an adequate choice independently. On its own, processing emotional experiences can induce adaptive and healthy desires; however, using compassionate self exercises helps the client to integrate complicated emotions and to approach their own values in a more direct way.

## 1. Introduction

Emotions are one of the most important subjects in psychotherapy. In psychodynamic therapy, emotions toward other people are referred to as transference, which is a key concept of psychoanalysis, and interpreted as emotions which should be directed toward caregivers [1]. In cognitive behavioural therapy, emotions also are a necessary means for treating cognitions [2]. In Compassion Focused Therapy (CFT) known as a new psychotherapy for shame and self-criticism [3, 4], emotional processing assumes an important role. In CFT, the therapist uses its unique model of emotions to help clients to understand their emotions [5, 6]. From the evolutionally perspective, emotions are regarded as survival functions and clients are encouraged to understand the function of each emotion [7, 8].

Understanding the means and influences of emotions can help clients to access their desires underlying each emotion and to shift to a compassionate state that will motivate them to choose the best way for themselves. Emotions are rooted in basic needs or desires; therefore, experiencing emotions can help clients to change their problematic emotional states and enable them to endure and address their emotions in a healthy way.

To help therapists to understand client's emotions, emotions are separated into four types: adaptive primary emotions, maladaptive primary emotions, secondary emotions, and instrumental emotions in Emotion Focused Therapy (EFT) [9, 10]. Among these emotions, adaptive primary emotions and maladaptive primary emotions are responses to a stimulus or the environment. Adaptive primary emotions are natural responses for survival in dangerous situations. Therefore, in a crisis, adaptive primary emotions often present as anger, fear, or anxiety, and represent a healthy response to the situation. On the other hand, maladaptive primary emotions are learned from past events and may include shame, feeling unloved or worthless, sadness resulting from loneliness or deprivation, and anxiety resulting from feeling inadequate or insecure. As debilitating fear and anxiety, shame and humiliation, destructive rage, and unresolved grief are main contenders of maladaptive primary emotions, these emotions can be difficult to regulate or manage [9]. As a result, people tend to ruminate about maladaptive primary emotions and related thoughts.

The phenomenon of experiencing maladaptive primary emotions can be regarded as an uncontrolled activation of the threat system, which is one of the motivational and emotional systems in the context of CFT. CFT theory emphasises the importance of soothing when the emotional response related to threat is activated [5]. The threat system is based on a protective function to survive and presents as fear, anxiety, anger, or disgust, which can function as both adaptive and maladaptive primary emotions depending on the situation.

However, there are commonalities in the approach to such threat emotions in CFT and EFT, such as soothing emotions and validation from others. In CFT, therapists need to be compassionate toward the client's experiences and demonstrate validation and warmth. Such attitudes can help clients to face painful emotions and shift to recognize the desires underlying these emotions, and they can work in a soothing function to regulate the threat system [5]. In EFT, soothing emotions are also emphasised and are achieved by internalizing the therapist's empathy or validation for the painful experiences [9, 10].

In addition, to understand complicated emotions such as maladaptive primary emotions, CFT therapists use the concept of multiple selves [11]. The multiple selves are based on the assumption that our mind consists of many version of the self [12]. For example, an individual consists of parts that are angry, anxious, fearful, disgusted, joyful, and so on. All these parts contribute to one's perception of self and one's experiences. Based on this assumption, individuals may experience a confused state of maladaptive primary emotions in which they have not experienced each emotion fully and are instead dominated by mixed emotional pain. Thus, separating each emotion from the mixed emotional pain and experiencing an emotion individually can help clients to address the desires underlying the emotions, which in turn leads them to cope in a healthy way.

Furthermore, a compassionate self has wisdom, kindness, confidence, and courage, which can be a compass to guide one toward one's own values like the captain of a ship [12]. The compassionate self can listen to each version of the self and form an integrated opinion of oneself. In CFT, adding to the validation for the client's experience and demonstrating empathy in a compassionate way, the therapist helps the client to integrate their desires and decide how to react to problems and pain by using the client's compassionate self.

This case report provides an example of a client with complicated maladaptive primary emotions. Therapy involved emotional processing and generating a compassionate attitude toward the problem through the therapist's validation, as well as the use of empathetic response, multiple selves, and compassionate self exercises. The case report was approved by the ethical committee of the graduate school of medicine, Chiba University.

## 2. Case Presentation

### 2.1. Case

Julia initially applied to the research program of a clinical trial of group compassionate mind training (CMT) for treatment of residual depression. She was assessed based on study inclusion and exclusion criteria and met the criteria for enrolment. For participation, she brought a referral letter from her psychiatrist which stated that she appeared to have the ability to participate in a group format. To assess her symptoms, including the inclusion and exclusion criteria, the Japanese version of the Beck Depression Inventory II (BDI-II) [13, 14], the Japanese version of The GRID-Hamilton Depression Rating Scale (GRID-HAMD) [15, 16], and the Japanese version of the Mini-International Neuropsychiatric Interview (M.I.N.I.) [17, 18] were administered. She scored 34 points on the BDI-II and 22 points on the GRID-HAMD, which indicated the presence of severe depressive symptoms. She also fulfilled the criteria for social anxiety disorder and generalised anxiety disorder on the M.I.N.I.. At the beginning of the group sessions, she appeared anxious, but she was able to discuss those feelings in the first session. When she discussed her painful experiences related to bullying, maltreatment, and isolation in school, other group members and the therapist validated her emotional suffering. At first, she argued that those experiences were caused by her fault and flaws. However, over time, validation from the group members and psychoeducation regarding CMT appeared to help her to accept her own emotional responses. She began to express anger toward the people who hurt her and to gradually engage in social activities. After treatment in the group, she scored 22 points on the BDI-II, indicating moderate depressive symptoms; however, she reported partial improvement when providing free writing feedback. As she hoped for remission of her symptoms and to promote social rehabilitation, she planned to receive individual sessions with charge.

### 2.2. Presenting Problems

Julia complained that she had trouble with her relationship with her parents and stated that she hoped to work in the future. She had started to go out to get used to being outside as part of graduated exposure. Regarding family relationships, she was often criticised for her behaviours at home, such as resting. Furthermore, if she expressed anxiety, her family members did not validate her feelings.

### 2.3. Background and History

She was maltreated as a child and was frequently compared to her sister and told “you are not cute” or “you are a fool” by her mother. Unlike her mother, her father did not attack her, but he also did not provide help. She experienced terrible bullying in primary school which continued into junior high school. In high school and university, she was not bullied, but she had difficulty building relationships with friends. After graduating university, she looked for work but was unable to find a job. Her parents began to talk only about her unemployment, and she presented to a psychiatric clinic due to depression and panic attacks. After receiving group CMT as described above, she began to go out and her panic attacks disappeared.

### 2.4. Formulation

**Figure 1:**
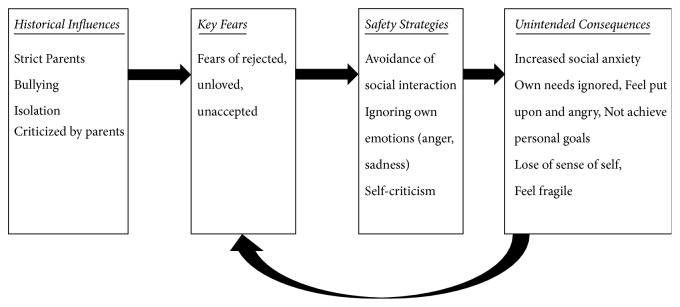
Case formulation of CFT adapted from Kolts et al.'s work [19].

The case formulation is displayed in Figure 1 [19]. As mentioned above, Julia had experienced maltreatment from her parents and bullying and isolation in school. Such experiences led her to fear rejection and feel unloved and unaccepted. To avoid her key fears, she stopped going out, ignored her feelings of anger and sadness, and criticised herself when she did not meet her parent's standards. Use of safety strategies induced an increase in anxiety and loss of a sense of self. As a result, she ignored her own needs and key fears were strengthened.

Based on this case formulation, validation for her emotional response was thought to be important. Receiving empathetic responses from others would help her to recognize and accept her own painful experiences and emotions. In addition, encouraging her to engage in social activities was thought to be effective in helping her achieve her goals. As she began to have new interactions with others, she would likely experience anxiety and resistance to relationships; however, such activation of anxiety and resistance is necessary for recovery. As a result, the therapist planned to promote social activities to distance her from her parents who attack her, and encourage her to learn age-appropriate social skills to help her mature. Social situations also provide opportunities for her to reconsider her safety behaviours and acquire coping strategies in nonharmful relationships.

### 2.5. Intervention

The individual sessions were held once every two weeks. In the first individual session, Julia's plan to go out was discussed. As Julia hoped to acquire PC skills, she planned to go study at a library and café. In the second session, she complained that she was confused and nauseous. In this section, we demonstrate an example of emotional processing and compassionate attitude by introducing the log from the second session.

(Cl.) I am struggling with a problem. My stomach feels tight because I am so nervous. I received a message from a friend who was a classmate from high school. One of our friends will get married, so she contacted me to ask me to prepare a message card and photo of me for the celebration. However, I was betrayed by her before. I had thought that we had broken off a relationship when we were in high school. In fact, we didn't have any contact for a long time. She is a friend who betrayed me.
(Cl.) Of course, I would like to celebrate a friend who will get married; however, it is difficult for me to find the words except for “congratulations.” There is only one time we met after graduation from university. I can't feel anything. A friend who betrayed me said to me that “you are too heavy.” So, I decided to distance myself from her. We met again in the university, but we didn't have any chance to talk. There was a time when I sent her message to ask her advice, but she did not reply at all. So, I thought that she would not like to see me, she would like to refuse me, and I blocked her in social networking services. It was email from her, and I am so confused and in distress. In the email, she wrote “Let's get together! We are living nearby!” I can't imagine what she is thinking and feeling. She is so selfish. I think I can meet her. But, I won't find any words when I met her (Cl. showed therapist messages).
(Cl.) I don't know how she is feeling in sending this email. This is the first email from her. I replied that “She is going to get married, isn't she? The messages of celebration will be read in the wedding party?” After that, she sent me “Let's play together.” I can't imagine her intention. I received the email suddenly, she has already gotten married and had children, but I didn't know about it at all. It is very complicated for me.
(Th.) *How do you feel now?*
(Cl.) I feel nauseous. I can't imagine what she is thinking.
(Th.) *Disgust?*
(Cl.) Uh… I can't imagine what is her intention. But, if I could behave like an adult, we might be able to mend the relationship. However, I wonder is that my hope? Actually, we are living in the nearby city. But, I will be able to find only the words “Your baby is so cute.” I can't talk about my job, because I am not working. Of course, she is not familiar with depression, she said that the “Gloomy mood is hard for you.” I think she doesn't know about depression or mental problems.
(Th.) *Are you feeling anger?*
(Cl.) I don't know. I can't imagine anger. I can't understand anger now. I think it doesn't make any sense what she is doing. So, I feel nauseous, and have a stomach ache.
(Th.) *Does it feel like a disagreement in a quarrel, or you are saying that you can't really understand or imagine?*
(Cl.) It might feel like disagreement and anger. I'd like to say “What are you saying?! ” You ignored my messages, what's happened after marriage? I don't know what she is saying. Why she can say so, I don't know how to reply. I don't know. I am feeling strong nausea from yesterday. I can't imagine what should I do.
(Th.) *Are you remembering painful memories?*
(Cl.) Yes. We had a good relationship in high school. She was my best friend. But, after the separation of classes, she began to refuse me suddenly. I teared a letter from her. I think it was difficult for her to maintain our relationship, because we were in different classes. But, it is a painful memory for me and I have unbearable feelings. I have mixed feelings. I am feeling anger and would like to say, why could you change? You didn't know my pain, did you?
(Th.) *You were so hurt. You think “why?” *
(Cl.) Yes. I can't understand her.
(Th.) *You said “complicated”, can you feel other emotions?*
(Cl.) I wonder… it may be anger and I hope to resolve it in my heart. But, I don't hope to meet her, so I am confused.
(Th.) *I think it is natural for you to be angry and not to hope to meet again.*
(Cl.) Yes, I don't want to meet her again. On the other hand, I wonder if I should repair the relationship and make friends.
(Th.) *Do you mean that you would like to forgive her, or you should have more friends or relationship with others?*
(Cl.) I think I should make friends for my recovery from illness. If possible, it is better for me to repair the relationship, but it is difficult for me to forgive, because I was hurt. I also know it is better for me to make more friends.
(Th.) *You have multiple-selves, Yourself with anger, yourself with hope to go along with her, and yourself recommend you to have more friends for recovery.*
(Cl.) Yes, my adult part thinks that I should have more friends, and my child part is struggling with pain and anger. My nausea might be rooted in the gap between them. I also think that if I could repair the relationship, I wonder if it isn't good for me. I don't absolutely hope to repair the relationship with her, there is part of me who thinks it is not necessary for me.
(Th.) *You are really having complicated feelings, and your head is spinning. Would you remember the program of CFT? Angry self, anxious self, sad self, and so on, there are many aspects of self. The compassionate self is a kind of coordinator or leader who can listen to their feelings. She can hear angry feelings, and the idea of recommendations to make friends. Would you try to ask compassionate self in an exercise?*
(Cl.) Yes, I'd like to try it. I can feel anger and hope to forgive. But, I was not invited to the party… I am feeling anger again, why must I send a message without an invitation. If I asked about it to compassionate self, she might recommend me to send a photo and message, but what should I do about the relationship… I feel it does not matter for me to repair. However, I am happy to be asked to play. I had experienced painful bullying. When I was asked to play, I felt that “I am needed by others” and felt so happy. But, the anger is stronger than it now. Why now?
(Th.) *You might be re-experiencing painful memories at that time again.*
(Th.) *Would you try the exercise to help yourself?*
(Cl.) Yes.
(Th.) *Can you imagine the compassionate self? Please imagine someone who has compassion for you and ask how to cope with this problem (*Conducting Soothing Rhythm Breezing and Compassionate Self exercise).[3]
(Th.) *How do you feel?*
(Cl.) I am feeling relaxed. I feel I should do what I need to do. The compassionate self recommended for me to send a photo and message. I will feel sorry for the absence of a message, I need to send it. However, when I talked to myself with the painful memory, she did not hope to repair the relationship and did not hope to meet the friend in high school again. I'd like to send the photo and message, but not to repair the relationship purposely.
(Th.) *She gave you good advice, you might have a chance to hope to re-connect with them in the future. I think it is important for you to be compassionate to yourself now.*
(Cl.) Yes, I'd like to say “see you again.”
(Th.) *You are challenging [yourself] to other social tasks like studying or working in a part-time job. I think you are doing better than you think now. How do you feel now?*
(Cl.) I am feeling good. It is hard to believe I had nausea.
(Th.) *It was a very difficult event for you.*
(Cl.) Yes! I was very surprised. I thought why do you know my email address?
(Th.) *I wonder if you might feel difficulties in thinking about or sending the message. If you feel difficulties, please ask the compassionate self how to cope with it. She would be a friend and support you. I felt your compassionate self is dependable.*
(Cl.) Yes. I noticed that pain does not disappear easily.
(Th.) *Yes. So, you don't need to pretend that nothing happened, but you can help yourself as you did now.*

## 3. Discussion

At first, Julia complained about her current problem which was caused by an email from an old friend. She was distressed and felt stomach pain and nausea. Such distressed states were caused by her painful experiences in the past. The therapist tried to help the client to identify her mixed emotions by asking questions about what she was feeling. Negative emotions have a function for blocking emotional processing; however, awareness of one's own emotion can weaken its impact [20]. In this scene, the therapist helped Julia to become aware of her own emotion and it led her to process her emotional state. In other words, these questions worked as a facilitator to process her emotional experience which led her to distinguish anger from the other mixed emotions. The therapist helped the client to shift her attention to emotions and emphasised her statements to induce her emotional response. Such methods are used in EFT to facilitate access to adaptive emotions and needs [9]. The therapist also validated her emotional response by citing her painful memory to facilitate her emotional process. As a result, she could regulate her own emotions and recognize her own desires (anger, the idea of having more friends, and hope to repair relationships) from the mixed emotional state. From the quantitative study, it was revealed that emotion regulation has a role in mediating the relationship between self-compassion and mental health [21]. Helping Julia's emotion regulation may be related to her exploring and accessing her internal compassionate self and led to the resolution of emotional pain.

However, these desires were still complex and remained confusing. After Julia identified and distinguished each emotion from her complicated feelings, the therapist introduced the multiple selves concept from the CFT exercises. The multiple selves exercise is a useful method to respect each aspect of the self by shifting attention from one aspect to another aspect one at a time. This helps the client to recognize not only each desire, but also the complicated and confused self in a mindful way. In addition, compassionate self can help clients to integrate all aspects of the self into one direction which would indicate the best decision. In this case, Julia was advised by the compassionate self of what to do in a way that was in alignment with her values. This phenomenon is similar to reports that revealed the relationship between self-compassion and well-being or self-compassion and psychopathology [22, 23].

In many cases, clients are suffering from mixed and confused emotional states, usually consisting of maladaptive emotions. To recognize and experience emotions in an adequate way can help such clients to process and sort out their emotions. The therapist needs to demonstrate empathy and understanding regarding their mixed and confused states and help them to pay attention to and process emotions in many ways. Emotional processing can lead the desires underlying emotions to the client's subconscious and direct clients to make a healthy choice in alignment with one's own values.

If the client has multiple desires, they can be thought as multiple aspects of the self. Each aspect has its own reasons and contexts, and therapists need to help clients to respect and understand each aspect of their desires. Understanding each aspect is a kind of cultivation of the compassionate self; therefore, noticing multiple selves can be the foundation of the compassionate self. The compassionate self can work as a captain of a ship who leads the crew (selves) to the best decision for all selves. The compassionate self helps the client to choose realistic and appropriate ways to integrate the client's multiple concerns.

## 4. Conclusion

In conducting CFT, paying attention to emotions and desires is necessary and EFT techniques to process emotional experiences are useful. Moreover, compassionate self exercises can play a role in integrating confused or complicated emotions, to direct clients to adequate choices independently. Processing emotional experiencing can induce adaptive and healthy desires alone; however, using compassionate self exercise helps the client to integrate confused or complicated emotions and to more directly approach their own values.
